# Effect of the Pressure of Reaction Gases on the Growth of Single-Crystal Graphene on the Inner Surfaces of Copper Pockets

**DOI:** 10.3390/mi11121101

**Published:** 2020-12-14

**Authors:** Kaiqiang Yang, Jianlong Liu, Ruirui Jiang, Yubin Gong, Baoqing Zeng, Zichuan Yi, Qingguo Gao, Jianjun Yang, Feng Chi, Liming Liu

**Affiliations:** 1 School of Electronic Science and Engineering, University of Electronic Science and Technology of China, Chengdu 610054, China; 201811022515@std.uestc.edu.cn (K.Y.); liujianlong@uestc.edu.cn (J.L.); 201711040120@std.uestc.edu.cn (R.J.); ybgong@uestc.edu.cn (Y.G.); 2 Zhongshan Branch of State Key Laboratory of Electronic Thin Films and Integrated Devices, University of Electronic Science and Technology of China, Zhongshan Institute, Zhongshan 528402, China; yizichuan@zsc.edu.cn (Z.Y.); gqg@hust.edu.cn (Q.G.); sdyman@uestc.edu.cn (J.Y.); chifeng@semi.ac.cn (F.C.)

**Keywords:** single-crystal graphene, copper pocket, growth pressure, nucleation density, size

## Abstract

Single-crystal graphene has attracted much attention due to its excellent electrical properties in recent years, and many growth methods have been proposed, including the copper pockets method. In the copper pockets method, a piece of copper foil is folded into a pocket and put into a chemical vapor deposition (CVD) system for the growth of graphene. The dynamic balance of evaporation and deposition of copper on the inner surfaces of the copper pockets avoids high surface roughness caused by the evaporation of copper in open space, such as the outer surfaces of copper pockets. Much lower partial pressure of methane in the copper pockets and lower surface roughness reduce the nucleation density of graphene and increase the size of single-crystal graphene. It is found that the growth pressure is closely related to the size of single-crystal graphene prepared by the copper pockets method; the higher the growth pressure, the larger the size of single-crystal graphene. It is also found that the growth pressure has an effect on the inner surface roughness of the copper pockets, but the effect is not significant. The main factor affecting the size of the single-crystal graphene is the change in the volume of the copper pockets caused by the change in the growth pressure, and the volume of the copper pockets determines the content of methane in the copper pockets. According to the above law, the size of single-crystal graphene prepared by the copper pockets method can be enlarged by increasing the growth pressure. The size of single-crystal graphene can be enlarged in a wide range as the growth pressure can be increased in a wide range. In our experiments, when the growth pressure reached 450 Pa, single-crystal graphene with a diameter of 450 μm was prepared.

## 1. Introduction

Graphene, the thinnest carbon material with atoms arranged in a two-dimensional hexagonal lattice, was first isolated by mechanical exfoliation from highly oriented pyrolytic graphite (HOPG) and has exceptional chemical stability, superior mechanical stability, very high thermal conductivities and electronic conductivities [1,2,3]. Because of the above excellent properties, graphene has potential applications in ultra-high-speed electronics [4], flexible transparent conductive films [5,6], solar cells [6], separation membranes [7], transmission electron microscopy (TEM) imaging [8], and biomedicine [9]. To date, the best-quality graphene has been obtained by mechanically exfoliating highly oriented pyrolytic graphite [1]. However, this method is limited by poor scalability considering the limited size of graphene flakes and the difficulty in carefully controlling the number of layers. Various methods to synthesize graphene have been developed to address these challenges, among which chemical vapor deposition (CVD) has emerged as the most promising technique owing to its capability to provide high-quality, large-area production, controllability of the number of layers, and good repeatability [10]. The CVD method for graphene synthesis was first reported in 2006 on Ni substrate [11]. In the aspect of substrate selection, Cu is considered as one of the best metal substrate materials because of its low carbon solubility, well-controlled surface, and low cost. A high-quality single-layer graphene film was successfully synthesized on Cu for the first time in 2009 [12]. However, since the day the CVD method appeared, there are still lots of challenges, such as controlling the number of graphene layers, minimizing wrinkling, and increasing the size of single crystals [13].

Grain boundaries produced during material synthesis affect both the intrinsic properties of materials and their potential for high-end applications. This effect is commonly observed in graphene film grown using chemical vapor deposition. Large-area graphene films are generally merged from small single-crystal domains and, therefore, have lattice defects, which result in challenges in the consistency of graphene materials. Based on this, the performance of graphene devices is uneven, and accordingly, it is necessary to study the growth and preparation of single-crystal graphene. Therefore, the growth of single-crystal graphene has aroused great interest in recent years. Graphene grain boundaries (GGBs), which form in regions where graphene domains with different orientations merge, are observed as line defects in graphene film and result in degradation of the thermal, electrical, and chemical properties of graphene [14,15,16,17,18,19]. For example, the electrical performance of graphene is degraded by the additional scattering centers induced by GGBs, causing a reduction in the carrier mobility and conductivity of graphene [20].

There are two ideas to improve the size of single-crystal graphene, which are the single-seed method and the multi-seed method. The key of the single-seed method is to control the nucleation density of graphene. It is found that higher surface roughness and impurity pollution will increase the nucleation density because these factors will increase the number of active sites on the surface of the substrate and then increase the nucleation probability of graphene [21,22]. Therefore, it is necessary to pretreat the substrate surface; ex situ pretreatment methods include surface rinsing, etching, and polishing [23,24]. Another method to control the nucleation density of graphene is the control of carbon source supply during graphene growth. Lower carbon supply will also reduce the nucleation probability of graphene [25,26,27]. The multi-seed method requires the preparation of special substrates. The orientation dependence of graphene on the substrate surfaces, which is vital to the aligned nucleation of graphene islands, is mainly determined by the interaction and the lattice matching degree between graphene and underlying substrates. To date, epitaxial growth of graphene has been reported on the surfaces of some special substrates, such as Cu(111), Ni(111) [28], Au(111) [29], Pt(111) [30], and CuNi alloy [27,31]. Among these, the Cu(111) plane is highly advantageous for aligned nucleation and growth of graphene domains as its hexagonal lattice symmetry matches the honeycomb lattice of graphene well (lattice mismatch ≈ 4%), thereby enabling epitaxial growth of graphene, on which the graphene islands can be simultaneously grown and seamlessly merged [32,33,34]. Based on the two ideas, many methods have been proposed to prepare single-crystal graphene in recent years, including the copper pockets method. In 2011, Li and co-workers obtained single-crystal graphene with an edge size of up to 0.5 mm, for the first time, on the inner surfaces of copper pockets. Copper pockets were employed as the substrate rather than simple copper foils in their experiments, and the graphene grew at a very low pressure with a slightly lower methane flow rate. The successful growth of large domains in the enclosed spaces was rationalized as the improvement of the growth environment, including the much lower partial pressure of methane and a much lower density of nuclei, followed by a long period of surface-growth along the edge of the nuclei atoms. In addition, the multibranched or dendritic features of the obtained graphene domains indicate that the growth is a typical diffusion-limited process [35], unlike in typical low pressure chemical vapor deposition (LPCVD) in an open reaction space, where the surface reaction regime controlled the growth process. The transferred graphene films showed a mobility of greater than 4000 ${\mathrm{cm}}^{2}\cdot V^{-1}\cdot s^{-1}$, indicating high crystalline quality [36]. After this pioneering work, Chen and co-workers proposed a Cu evaporation suppression mechanism based on three types of space-confined strategies. Their explanation for how millimeter-scale single-crystal graphene can grow on the inner surfaces of these kinds of structures is that the process of evaporation and deposition of copper on inner surfaces tends to be stable at high growth temperatures, and a smooth inner surface will be obtained [37,38]. It was reported that the number of active sites can be reduced by using smooth substrates [22].

In this paper, we use the copper pockets method to prepare single-crystal graphene. It is found that the growth pressure has a positive correlation with the size of the single-crystal graphene, and the mechanism is discussed. In our experiments, single-crystal graphene with diameter of 450 μm can be prepared at 450 Pa. Unlike reduced graphene oxide (rGO) and polycrystalline graphene, single-crystal graphene is highly impermeable, conductive, and strong owing to its nearly perfect lattice. The single-crystal graphene prepared by us can be used in the preparation of flexible electrodes [39]. Due to the high carrier mobility of the single-crystal graphene, it can also be used for the preparation of a flexible field effect transistor (FET) [40].

## 2. Materials and Methods

### 2.1. Synthesis of Graphene

Graphene was synthesized by low-pressure CVD in a tube furnace. As shown in Figure 3b, a Cu pocket was made by first bending a copper foil (Graphene Technology company in XiaMen, China; purity of 99.8% and thickness of 25 μm) and then crimping or pressing the three open edges carefully by using metal tweezers. During the fabrication, no special mechanical machine was used to seal the edges [41]. The copper pockets were placed flat in the quartz boat, which was located in the center of the quartz tube of CVD system. Then, the CVD system was vacuumed to 0.3 Pa, and 50 sccm $H_{2}$ was introduced for 10 min to drain the remaining air in the quartz tube. The temperature of the CVD system was raised to 1030 ℃ within 30 min, after which the copper pockets were annealed with 100 sccm Ar for 65 min. ${\mathrm{CH}}_{4}$/$H_{2}$ mixed gas with a 1/50 flow ratio was introduced at the growth stage, and the growth pressure was adjusted to 3, 50, 300, and 450 Pa by adjusting the knob of vacuum pump. Finally, the temperature of the CVD system was reduced to room temperature and the flow rates of ${\mathrm{CH}}_{4}$ and $H_{2}$ were maintained.

### 2.2. Transfer of Graphene

A thin film of polymethyl methacrylate (PMMA) (HF-kejing, 6 wt.% in anisole) was spin-coated onto the graphene/Cu (600 r/min for 6 s then 3000 r/min for 40 s). The etching of Cu usually takes 3 h. Then, the PMMA/graphene/Cu thin film was put into 30 mg/mL ammonium persulfate ((NH_4_)_2_S_2_O_8_) aqueous solution (Aladdin, purity of 98%, AR). After etching, the PMMA/graphene was rinsed in DI water several times. The PMMA/graphene was dried in a desiccator and then placed on the SiO_2_/Si substrate. The PMMA layer was dissolved using hot acetone.

### 2.3. Characterization

After finishing the growth of graphene, the copper pockets were opened to copper foils and the copper foils were placed onto a hot table at 200 ℃ for 5 min. The oxidized surfaces of copper foils were observed under an optical microscope. Due to the oxidation resistance of graphene [42], the areas covered by graphene will not be oxidized, and the areas not covered with graphene will be oxidized to copper oxide. The areas covered by graphene were brighter than other areas. Therefore, we could directly observe the shape and size of single-crystal graphene on the copper foils with an optical microscope. An atomic force microscope (AFM) (Bruker, EDGE, Peabody, MA, USA) was used to acquire the inner surface morphology images of the copper pockets, and the corresponding surface roughness values were calculated.

## 3. Results and Discussion

**Figure 1:**
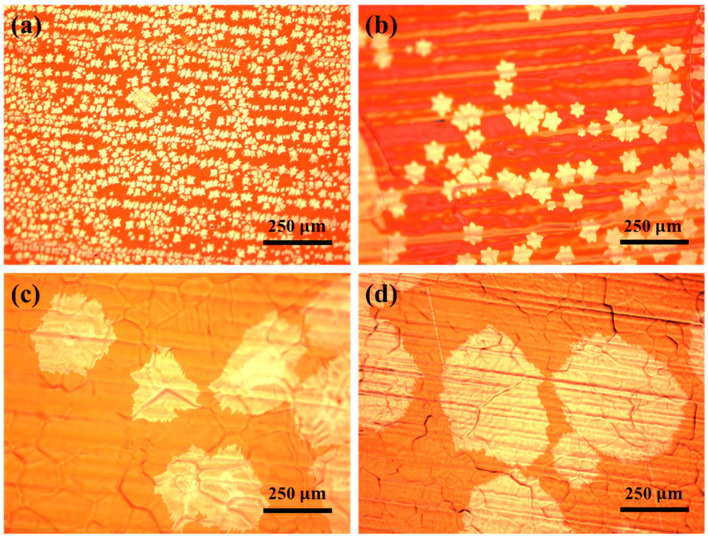
Optical micrographs of single-crystal graphene grown at different growth pressures under 200-times magnification observation: (**a**) 3, (**b**) 150, (**c**) 300, and (**d**) 450 Pa.

The single-crystal graphene grown on the inner surfaces of copper pockets in the low-pressure environment presents a fractal structure resembling a snowflake [36]. Figure 1 shows the optical micrographs of single-crystal graphene grown at different growth pressures. Figure 1a–d are optical micrographs of single-crystal graphene grown at 3, 150, 300, and 450 Pa under 200-times magnification observation, respectively. It is shown that the size of single-crystal graphene increases and the nucleation density of graphene decreases with the increase in the growth pressure.

**Figure 2:**
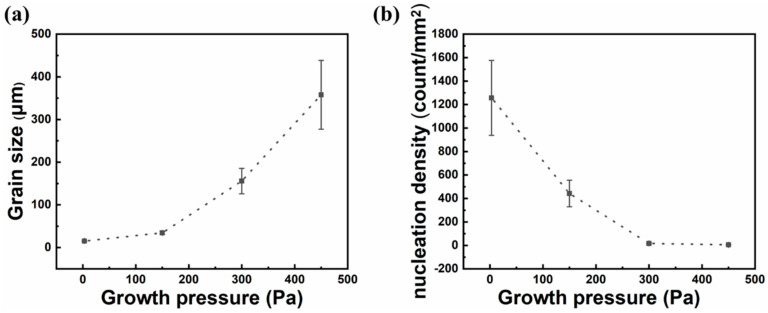
Nucleation density of graphene and size of single-crystal graphene at 3, 150, 300, and 450 Pa. (**a**) Relationship between growth pressure and size of single-crystal graphene. (**b**) Relationship between growth pressure and nucleation density of graphene.

**Figure 3:**
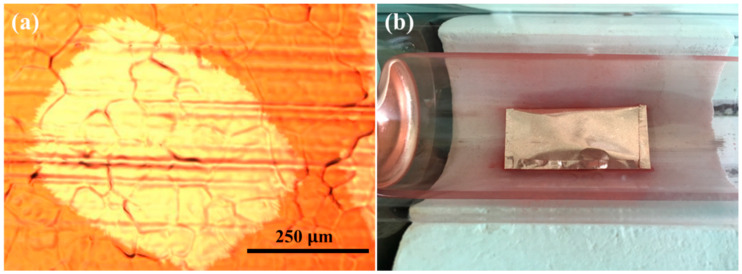
(**a**) An optical micrograph of single-crystal graphene with a diameter of 450 μm under-200 times magnification observation. (**b**) The physical object of the copper pocket.

Figure 2 shows the size of single-crystal graphene and the nucleation density of graphene at different growth pressures. It is obvious that the size of single-crystal graphene is positively correlated with growth pressure, and the size of single-crystal graphene increases with the increase in growth pressure. When the growth pressure is 150 Pa, the size of single-crystal graphene is 34 μm. When the growth pressure reaches 300 Pa, the size of single-crystal graphene is 156 μm. As shown in Figure 3a, single-crystal graphene with a diameter of 450 μm can be prepared when growth pressure reaches 450 Pa. On the other hand, the nucleation density of graphene is negatively correlated with growth pressure. It can be seen that with the increase in growth pressure, the nucleation density of graphene decreases. When the growth pressure is 3 Pa, the nucleation density of graphene is as high as 1257 per square millimeters. When the growth pressure reaches 300 Pa, the nucleation density of graphene decreases to 17 per square millimeters. When the growth pressure reaches 450 Pa, the nucleation density of graphene can reach 5 per square millimeters.

The nucleation density of graphene is closely related to the surface roughness of the substrates. The number of active sites on the uneven surface was higher, which increased the nucleation probability of graphene. Active sites are generally reported to be sites with higher surface roughness or covered by impurity or contamination on the surface [43]. Chen and her team were inspired by this point in their explanation for the principle of preparing single-crystal graphene with the copper pockets method in 2013. They found that when the copper in the inner surface evaporates, it redeposits on the inner surface and so no or little copper is lost (a small amount of loss of copper might occur at the open ends), and the inner surface was found to be much smoother than the outer surface in identical CVD growth conditions [37]. The dynamic balance of evaporation and deposition of copper inside copper pockets ensures the inner surface of the copper pockets smooth. Therefore, we further investigated the relationship between the inner surface roughness of the copper pockets and the growth pressure after observing that the nucleation density of graphene decreases with increase in growth pressure. The inner surface roughness of the copper pockets under different growth pressures was measured by atomic force microscopy (AFM).

**Figure 4:**
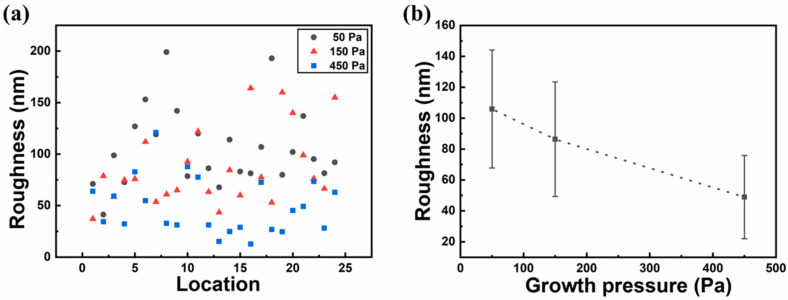
Statistical analysis of the variation of inner surface roughness of copper pockets with growth pressure. (**a**) The roughness values of 24 different locations on the inner surface of each copper pocket under 50, 150, and 450 Pa. (**b**) The relationship between growth pressure and the inner surface roughness of the copper pockets.

**Figure 5:**
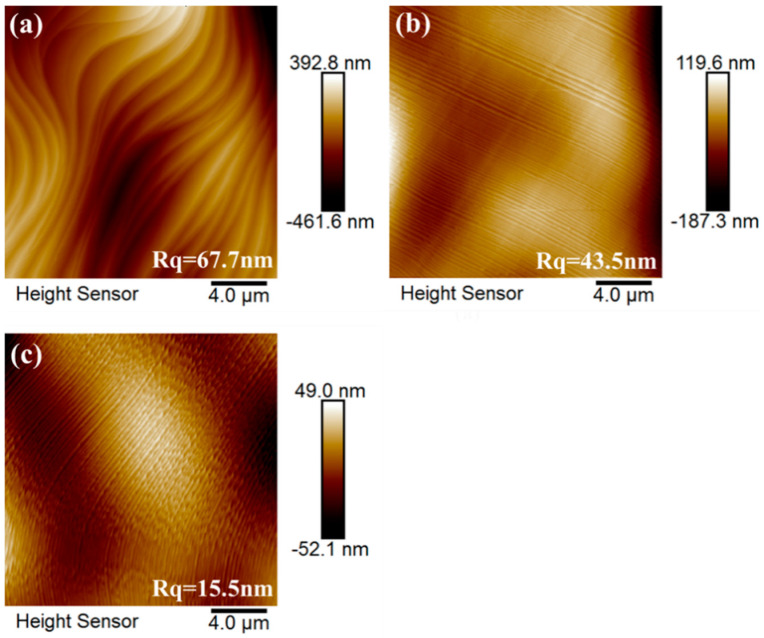
Atomic force microscope (AFM) images of inner surface of copper pockets at location 13 (**a**) at 50 Pa; (**b**) at 150 Pa; (**c**) at 450 Pa.

Figure 4a shows the roughness values of 24 different locations on the inner surface of each copper pocket under different growth pressures. Figure 5 shows AFM images of the inner surface of copper pockets in location 13 (at 50, 150, and 450 Pa). It can be seen from Figure 4a that when the growth pressure is 50 Pa, the inner surface roughness (black circle) of the copper pockets is roughly distributed in the range of 41.2–199.0 nm; when the growth pressure is 150 Pa, the inner surface roughness (red triangle) of the copper pockets is roughly distributed in the range 37.1–164.0 nm; when the growth pressure is 450 Pa, the inner surface roughness (blue square) of the copper pockets is roughly distributed in the range 12.8–121.0 nm. The distribution results of the inner surface roughness of the copper pockets at 50 and 150 Pa are very similar, but many of the roughness values at 450 Pa are lower than those at 50 Pa. Figure 4b shows the relationship between growth pressure and the inner surface roughness of the copper pockets. It is found that growth pressure will affect the inner surface roughness of copper pockets according to the statistical analysis, and the higher the growth pressure, the lower the inner surface roughness of copper pockets, but this effect is not strong. We believe that the change in external growth pressure has an effect on the establishment of a dynamic balance of evaporation and deposition of copper inside copper pockets, but this effect is relatively weak, which has little effect on the surface roughness of copper pockets.

**Figure 6:**
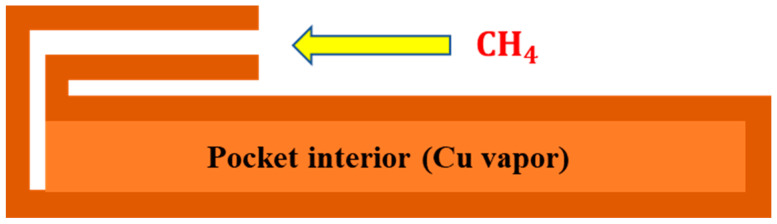
A schematic drawing of the cross-section of Cu pocket showing the ${\mathrm{CH}}_{4}$ transport channel into the pocket interior environment.

We also noted that the insides of the copper pockets are hollow. At the high temperature of 1030 ℃, the copper will evaporate and there will be vapor pressure inside the copper pockets. When the copper pockets are in the low-pressure growth system, the copper vapor pressure generated by evaporation of copper inside the copper pockets will separate the upper and lower sides of the copper pockets at a certain distance. In the environment of high temperature and low pressure, we assumed that the copper vapor and ${\mathrm{CH}}_{4}$ in the copper pockets are ideal gases. After a period of growth, the pressure both inside and outside the copper pockets reaches an equilibrium. At this time, the pressure inside and the growth pressure outside the copper pockets are equal, $P_{inside}=P_{growth}$. Figure 6 shows a schematic drawing of the cross-section of Cu pocket and shows the ${\mathrm{CH}}_{4}$ transport channel into the pocket interior environment. Hao and co-workers found the relationship between the number density of methane molecules in copper pockets and the time as (1) in 2016 [41].

(1) $$n_{{\mathrm{CH}}_{4}}\left(t\right)=n_{0}\left(1-\exp\left(-\frac{t}{t_{0}}\right)\right)$$

where $n_{{\mathrm{CH}}_{4}}$ is the number density of ${\mathrm{CH}}_{4}$ molecules; t is growth time; $t_{0}$ is the characteristic time; $n_{0}$ is the number density of ${\mathrm{CH}}_{4}$ molecules in copper pockets after system stabilization; $n_{0}$ and $t_{0}$ are both constants. After a period of time, the number density of ${\mathrm{CH}}_{4}$ molecules in the copper pockets tends to be a constant. Because the copper pockets are relatively closed, the ${\mathrm{CH}}_{4}$ partial pressure in the copper pockets is relatively low, and the pressure in the copper pockets is equal to the partial pressure of copper vapor in the copper pockets, $P_{Cu\;vapor}=P_{inside}$.

In each experiment, we used the same growth temperature, so the temperature $T_{growth}$ inside the copper pockets is a constant. The amount of copper $n_{Cu}$ (copper vapor in the copper pockets) is the final result after the dynamic balance of evaporation and deposition of copper is established. The value of $n_{Cu}$ should be related to the establishment of the dynamic balance, and once the dynamic balance is established, $n_{Cu}$ will remain constant during the growth process. According to the previous discussion, the change in growth pressure has little effect on the establishment of a dynamic balance of evaporation and deposition of copper inside the copper pockets. Therefore, the amount of copper $n_{Cu}$ (copper vapor in the copper pockets) is hardly affected by the growth pressure $P_{growth}$, and the $n_{Cu}$ is also constant throughout the experiment. According to the ideal gas equation of state, the internal pressure $P_{inside}=P_{Cu\;vapor}=P_{growth}$, $n_{Cu}$, the growth temperature $T_{growth}$, and the volume of the copper pockets $V_{Cu\;pocket}$ should satisfy (2).

(2) $$P_{growth}V_{Cu\;pocket}=n_{Cu}RT_{growth}$$

**Figure 7:**
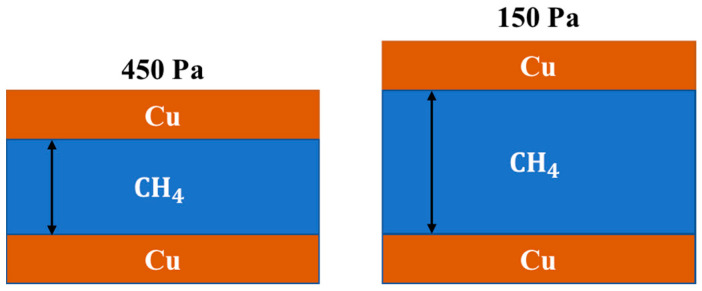
Variation in methane content with the growth pressure in copper pockets.

The volume of the copper pockets $V_{Cu\;pockets}$ is calculated from (3).

(3) $$V_{Cu\;pocket}=\frac{n_{Cu}RT_{growth}}{P_{growth}}$$

where R = 8.31 J/(mol·K), and because $n_{Cu}$, R, and $T_{growth}$ are constant, $V_{Cu\;pocket}$ is inversely proportional to $P_{growth}$. When the growth pressure increases, the volume of copper pockets is smaller. Carbon source supply during graphene growth is also an important factor affecting the nucleation density of graphene [27]. The decrease in the volume of copper pockets can determine the content of methane in the copper pockets. As shown in Figure 7, compared with 150 Pa, the volume of the copper pockets stretched by the copper vapor pressure at 450 Pa is smaller, so the content of ${\mathrm{CH}}_{4}$ in the copper pockets will also be reduced. The decrease in carbon source supply will reduce the nucleation density of graphene and increase the size of single-crystal graphene. We think that this is the main factor that affects the nucleation density of graphene on the inner surfaces of copper pockets.

## 4. Conclusions

In conclusion, growth pressure will affect the establishment of a dynamic balance of evaporation and deposition of copper in copper pockets when single-crystal graphene is prepared using the copper pockets method, and it will then affect the inner surface roughness of copper pockets. This effect has been shown, as the higher the growth pressure, the smaller the inner surface roughness of the copper pockets, but this effect is small. The growth pressure also affects the volume of the copper pockets. The larger the growth pressure, the smaller the volume of the copper pockets stretched by the copper vapor and the less ${\mathrm{CH}}_{4}$ the copper pockets can hold. Lower carbon supply reduces the nucleation density of graphene and increases the size of single-crystal graphene. Based on the above conclusion, we can increase the size of single-crystal graphene by increasing the growth pressure. At 450 Pa, single-crystal graphene with a diameter of 450 μm was prepared. The size of single-crystal graphene can still be increased by increasing the growth pressure. The single-crystal graphene prepared by us can be used in many kinds of 2D electronics, such as flexible electrodes and flexible field effect transistors, because of its ultra-high carrier mobility and excellent flexibility.
